# Metabolic and Mitochondrial Functioning in Chimeric Antigen Receptor (CAR)—T Cells

**DOI:** 10.3390/cancers13061229

**Published:** 2021-03-11

**Authors:** Ali Hosseini Rad S. M., Joshua Colin Halpin, Mojtaba Mollaei, Samuel W. J. Smith Bell, Nattiya Hirankarn, Alexander D. McLellan

**Affiliations:** 1 Department of Microbiology and Immunology, University of Otago, Dunedin 9010, Otago, New Zealand; josh.halpin@postgrad.otago.ac.nz (J.C.H.); smisa123@student.otago.ac.nz (S.W.J.S.B.); 2 Department of Microbiology, Faculty of Medicine, Chulalongkorn University, Bangkok 10330, Thailand; Nattiya.H@chula.ac.th; 3 Center of Excellence in Immunology and Immune-Mediated Diseases, Chulalongkorn University, Bangkok 10330, Thailand; 4 Department of Immunology, School of Medicine, Tarbiat Modares University, Tehran 14117-13116, Iran; mojtaba.mollaei@modares.ac.ir

**Keywords:** CAR T cell therapy, T cell metabolism, mitochondria, memory T cell, metabolic reprogramming

## Abstract

**Simple Summary:**

We review the mechanisms of cellular metabolism and mitochondrial function that have potential to impact on the success of chimeric antigen receptor (CAR) T cell therapy. The review focuses readers on mitochondrial functions to allow a better understanding of the complexity of T cell metabolic pathways, energetics and apoptotic/antiapoptotic pathways occurring in CAR T cells. We highlight potential modifications of T cell metabolism and mitochondrial function for the benefit of improved adoptive cellular therapy. Reprogramming metabolism in CAR T cells is an attractive approach to improve antitumour functions, increase persistence and enable adaptation to the nutrient-restricted solid tumour environment.

**Abstract:**

Chimeric antigen receptor (CAR) T-cell therapy has revolutionized adoptive cell therapy with impressive therapeutic outcomes of >80% complete remission (CR) rates in some haematological malignancies. Despite this, CAR T cell therapy for the treatment of solid tumours has invariably been unsuccessful in the clinic. Immunosuppressive factors and metabolic stresses in the tumour microenvironment (TME) result in the dysfunction and exhaustion of CAR T cells. A growing body of evidence demonstrates the importance of the mitochondrial and metabolic state of CAR T cells prior to infusion into patients. The different T cell subtypes utilise distinct metabolic pathways to fulfil their energy demands associated with their function. The reprogramming of CAR T cell metabolism is a viable approach to manufacture CAR T cells with superior antitumour functions and increased longevity, whilst also facilitating their adaptation to the nutrient restricted TME. This review discusses the mitochondrial and metabolic state of T cells, and describes the potential of the latest metabolic interventions to maximise CAR T cell efficacy for solid tumours.

## 1. Introduction

Generally, a CAR is composed of both antibody and T cell receptor (TCR) components that can recognise tumour-associated antigen (TAA) in an MHC-independent manner [1]. Indeed, this is the most significant advantage of CAR T cells over other types of adoptive cell therapy (ACT), such as tumour-infiltrating lymphocytes (TILs) and TCR therapies, especially in cases where tumour cells lose or downregulate MHC expression [1]. Regardless of the generation of CAR, a CAR is composed of three main parts: the extracellular domain (antigen recognition domain and hinge), a transmembrane (TM) domain and intracellular domain(s). The antigen recognition domain is a single-chain variable fragment (scFV) consisting of variable light (VL) and heavy (VH) chain regions of a monoclonal antibody (mAb). A flexible linker, usually made of glycine and serine repeats, separates the VL and VH. The hinge and TM are commonly derived from CD28, CD8α or IgG. So far, five generations of CAR T cells have been developed utilizing a different combination of costimulatory domains in their intracellular domains (CD28, CD137, CD134, etc.). For more information about CAR T cell design, see the recent reviews [1,2].

CAR T cell therapy involves the isolation and transduction of patient or allogeneic donor-derived T cells with a cancer-specific CAR, followed by around two weeks ex vivo expansion prior to administration to the patient [3]. During this period, the patient is normally conditioned with lymphodepleting chemotherapeutic drugs to reduce competition from endogenous T cells to allow lymphopenic expansion of transfused CAR T cells [3,4]. Thus, ex vivo expansion of CAR T cells differs from physiologic T cells expansion occurring during physiological T cell responses in several aspects [3].

The efficacy of CAR T cell therapy has been most impressive in treating B-cell lymphoma expressing CD19. Recent studies have reported response rates for B-cell non-Hodgkin’s lymphoma (B-NHL) and diffuse large B cell lymphoma (DLBCL) in the range of 50–80% [5,6,7]. The high rate of success in the treatment of B-cell lymphomas can be attributed to several reasons such as isolated antigen expression, manageable side effects and minimal impact of patient risk factors such as age, previous treatment or high international prognostic index (IPI) score used to determine patient risk group and prognosis for B-cell lymphomas [5,6,7]. Overall, this equates to CAR T cell therapy as an effective treatment of B cell lymphoma. However, this same efficacy has not been seen in CAR T cell clinical trials for solid malignancies (see Table 1) [8,9].

In contrast to bloodborne malignancies, solid tumours possess other properties that aid in tumorigenesis. Solid tumours develop an immunosuppressive microenvironment that antagonises the efficiency of the immune response. This occurs through physical factors such as low pH and immunosuppressive metabolites such as adenosine, but also through the increased expression of inhibitory ligands such as programmed cell death 1 (PD-1), cytotoxic T lymphocyte antigen 4 (CTLA-4) and lymphocyte activation gene 3 (LAG-3) [8,10]. In combination with the expression of inhibitory ligands, the extracellular environment is both highly acidic and hypoxic and being devoid of nutrients needed for cell survival and containing a high concentration of molecules that induce the recruitment and differentiation of immunoregulatory cell populations to maintain immune tolerance against the tumour [8,9,11]. For these reasons, current CAR T cell modalities have been ineffective in the treatment of solid tumours.

Therefore, several recent studies have focussed on ways to increase the efficiency of the T cell-mediated antitumour responses [9,12,13,14,15,16]. Effective antitumour responses require T cell infiltration to the TME, to allow T cell activation by tumour antigen recognition. T cell activation involves proliferation and differentiation, after which T cell subsets can carry out their various effector functions, which enable the destruction of malignant cells. Following rapid proliferation, T cells then undergo a contraction phase leaving long-lived memory T (T_M_) cells to maintain long-term protection from relapse. Metabolic regulation is critical to enable effective response [12,17]. T cells undergo significant metabolic reprogramming throughout activation to ensure energy requirements are met and providing biosynthetic pathways with sufficient intermediates to enable macromolecule synthesis of necessary cellular components [12,17].

Mitochondria play a key role in the regulation of T cell metabolism within the TME. The role of the mitochondria in T cells is multifaceted, with key roles in energy generation, biosynthesis, migration, cell fate and programmed cell death, all of which impact on the elimination of cancer [18]. T cells are exquisitely dependent on nutrient availability to carry out their effector functions and nutrient-sensing by T cells regulates their effector functions in accordance with the availability of nutrients in the surrounding environment [19]. The metabolically flawed nature of the TME has consequently been shown to have a severe impact on the efficacy of CAR T cell treatment of solid tumours and, therefore, patient prognosis [8,12,13,14].

## 2. Mitochondria Regulates Cellular Metabolism and Biosynthesis

Adenosine triphosphate (ATP) is a key metabolite for energy expenditure in cells. Three macromolecules are used to produce ATP, carbohydrates (e.g., glucose), fatty acids and amino acids (e.g., glutamine) by catabolic metabolism. Resting cells such as naïve T (T_N_) and T_M_ cells preferentially use this pathway to generate ATP [12,20]. Glucose catabolism initiates with glycolysis to generate two ATP and pyruvate.

**Figure 1:**
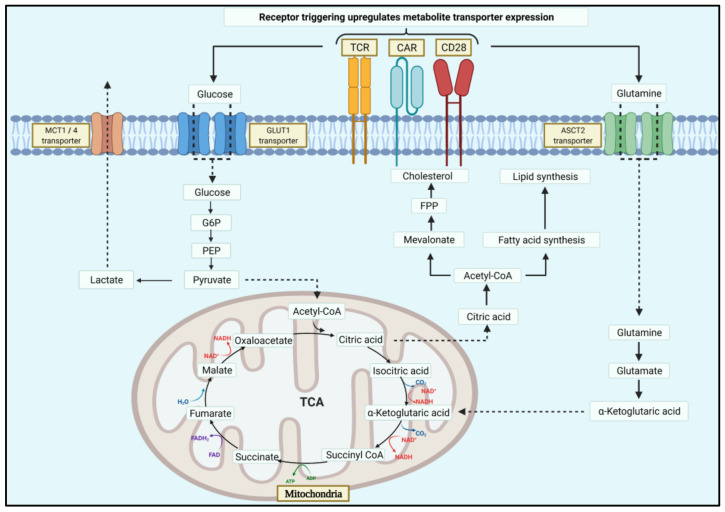
Schematic diagram of metabolic pathways in T cells. During T cell activation and rapid proliferation, glycolytic metabolism is enhanced by increases in glycolytic transporters such as GLUT-1. This increases glycolytically derived pyruvate conversion to acetyl-CoA, which is further converted to citrate within the TCA cycle. In the activated T cells, citrate is exported into the cytoplasm, where it is converted back to acetyl-CoA for use in the mevalonate pathway to synthesise cholesterol. To balance the export of citrate out of the TCA cycle, glutamine transporters are also upregulated. TCR, T-cell receptor; CAR, chimeric antigen receptor; MCT1, monocarboxylate transporter 1; GLUT1, glucose transporter 1; ASCT2, alanine-serine-cysteine transporter 2; G6P, glucose 6-phosphate; PEP, phosphoenolpyruvate; FPP, farnesyl diphosphate; TCA, Tricarboxylic acid cycle.

The tricarboxylic acid cycle (TCA) is a cyclical series of reactions used to generate additional ATP. The initial reaction involves the combination of acetyl-CoA, generated through fatty acid, pyruvate or amino acid oxidation, with oxalacetate resulting in the six-carbon citrate molecule. Citrate is then converted into isocitrate that is decarboxylated and converted to α-ketoglutarate, and then further to succinyl-CoA releasing two CO_2_ and one NADH molecule. Succinyl-CoA conversion to succinate is coupled with GTP synthesis, which can later be converted to ATP. Oxidation of succinate results in fumarate generation and the transfer of two H^+^ molecules transferring to FAD to produce FADH_2_. Fumarate is then converted to malate and further into oxalacetate to combine with acetyl-CoA to continue the cycle. NADH and FADH_2_ generate thirty-four ATPs by passing through the electron transport chain (ETC) [12,17,18,19,20,21] (Figure 1).

Rapidly proliferating cells such as effector T (T_EFF_) cells use anabolic metabolism, a process to manufacture new molecules to meet the demand for newly synthesised DNA, proteins and phospholipids for cell membranes. Acetyl-CoA produced in the mitochondria can be exported to the cytosol for the synthesis of fatty acids needed for lipid biosynthesis. Similarly, other metabolic intermediates from the TCA can be used for macromolecular synthesis, in a process known as cataplerosis, utilising cholesterol, nucleotides and amino acids needed for cell proliferation. This comes at the cost of metabolite depletion, in which the mitochondria balances through anaplerosis where molecules are fed back into the cycle. Anabolic metabolism only produces two ATP in exchange for one glucose through glycolysis [12,17,18,19,20,21].

## 3. An Overview of Mitochondrial Dynamic in T Cells

Mitochondria exist in two morphologies within T cells, either short or long tubules formed through fission or fusion [18]. Mitochondrial fusion and fission allow control over mitochondrial mass and metabolism. The mitochondrial dynamic is controlled by external (e.g., growth factors and nutrients) and internal factors (e.g., transcription factors and reactive oxygen species) [22]. The induction of these processes can be triggered in response to T cell activation and changes in T cells transcriptome [22], and environmental factors such as decreased nutrient availability, such that is seen in the TME [19] (see Section 5 and Section 6).

**Figure 2:**
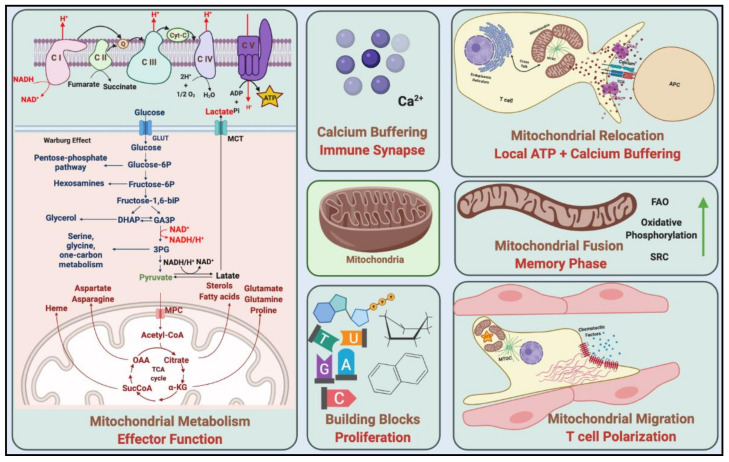
Mitochondrial functions in T cells. When T cells detect the chemotactic factors, mitochondria accumulate at the uropod to support the energy demand during T cell migration. Mitochondria take up calcium during stimulation and have a role in calcium homeostasis. Besides, mitochondria by anabolic metabolism provide ATP and energy for T cells, while by catabolic metabolism providing building blocks for cell proliferation. C I/II/III/IV/V, complex I/II/III/IV/V; MPC, mitochondrial pyruvate carrier; FAO, fatty acid oxidation; SRC, spare respiratory capacity.

In addition to ATP production, mitochondria are involved in lipid synthesis, signalling, calcium regulation and cell cycle progression, which all together act in an anabolic manner providing fundamental materials for the activation, clonal expansion and differentiation of T cells [18,21]. The contribution of mitochondria to these events includes fission and fusion, which fulfil adapting their location, size and distribution. There is also an interaction between mitochondria and other organelles, especially the endoplasmic reticulum, to maintain its function during the procedures mentioned above. These organisational structures are known as mitochondria-associated membranes (MAMs) [23]. During T cell adaptation, mitochondria travel towards the immune synapse, where they serve as local-ATP generators and calcium-buffering infrastructures to facilitate intracellular signalling and intercellular communication for T cell activation, proliferation and differentiation [18] (Figure 2).

The exact mechanisms of fusion and fission transition are poorly understood. In T cells, mitochondrial fission is triggered by T cell activation through the phosphorylation of dynamin-related protein 1 (Drp1) by protein kinase C. Drp1, a GTPase protein is translocated to the outer mitochondrial membrane (OMM) to fission sites by mitochondrial dynamic proteins, Mid49 and Mid51, and mitochondrial fission factor (MFF). Drp1 oligomerizes forming a belt around the mitochondria that constricts mitochondria through by GTP hydrolysis, splitting the inner- and outer-membranes [24,25].

Mitochondrial fusion involves three GTPases, mitofusion 1 and 2 (Mfn1 and Mfn2) and optic atrophy 1 (OPA1). During fusion, Mfn1/2 are localized at the OMM and form complexes between two adjacent mitochondria. C-terminal regions of Mfn1/2 contain hydrophobic heptad repeat region (HR2) that dimerizes with other Mfn1/2 proteins on the adjacent mitochondria bringing them into contact for fusion. OPA1 and cardiolipin are located within the mitochondria intermembrane space and are responsible for inner membrane fusion (IMM) [24,25].

Mitochondrial fusion increases cristae formation promoting increased oxidative phosphorylation (OXPHOS) and fatty acid oxidation (FAO); both pathways are essential for the survival of T_N_ cells and the formation of T_M_ cells [17,22]. In contrast, mitochondria fission promotes aerobic glycolysis within T_EFF_ cells, such as CD8^+^ T cells. This switch to anabolic metabolism is necessary to support the production of molecules needed to carry out effector functions [17,22].

## 4. The Role of Mitochondria in Cell Death

Cell death encompasses a variety of processes ranging from the relatively disordered necrosis to the highly ordered, active process of apoptosis. Regulation of cell death is crucial for maintaining homeostasis, at both the tissue and system level. Antigen specific activation of T cells typically leads to clonal expansion followed by contraction, with a small population of long-lived memory cells persisting in circulation [3]. A lack of T cell contraction leads to debilitating or even fatal lymphoproliferative disorders. Apoptosis can proceed through two distinct pathways, the intrinsic and extrinsic pathways, each of which has a distinct set of apoptotic cascades triggered by both overlapping and unique induction stimuli [26]. While both apoptotic pathways are triggered by distinct stimuli and follow divergent signalling pathways, they converge at the mitochondria, where the maintenance of mitochondrial membrane integrity acts as the essential mediator of apoptotic fate. Mitochondrial membrane integrity is maintained through the complex interplay of the Bcl-2 family of proteins, consisting of both pro-apoptotic (Bax, Bak, Bok, Bid, Bim, Bad, Noxa and Puma) and antiapoptotic members (Bcl-2, Bcl-xL, Bcl-w, A1 and Mcl-1) [26].

**Figure 3:**
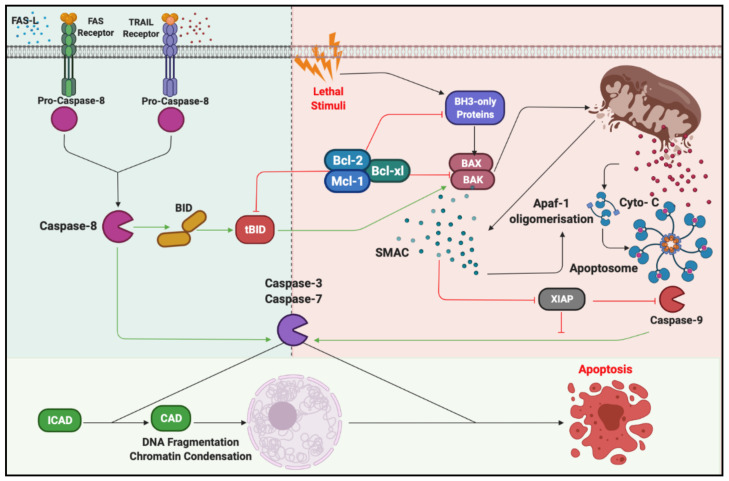
Mitochondrial pathways of cell death in T cells. Activation of CD95 or TNFR1 upon binding to CD95L results in the formation of DISC. Activation of caspase-8, in type I cells leads to direct activation of caspase-3 and then induction of apoptosis independent of mitochondria. In type II cells, caspase-8 cleaves the Bid proapoptotic protein to make truncated tBid. Next, tBid promote the oligomerisation of Bak/Bax complexes to form pores in the mitochondria outer membrane. Releasing Cyto-C in the cytosol activates caspase-9, which in turn activates caspase-3 to induce downstream events of apoptosis. TRAIL, TNF-related apoptosis inducing ligand; BID, BH3 interacting domain death agonist; CAD, caspase-activated DNase; ICAD, inhibitor of caspase-activated DNase; Bcl-2, B-cell lymphoma 2; Mcl-1, myeloid cell leukemia 1; Apaf1, apoptotic peptidase activating factor 1; Cyto-C, Cytochrome C; XIAP, X-linked inhibitor of apoptosis protein; TNFR1, tumor necrosis factor receptor 1; DISC, death inducing signaling complex.

Intrinsic apoptosis is triggered by a range of stimuli, such as DNA damage, oxidative stress or removal of homeostatic cytokines [27]. Under normal conditions, antiapoptotic members of the Bcl-2 family are able to sequester the function of the proapoptotic BH3-only family members. Apoptotic stimuli lead to increased BH-3-only protein activity, including the direct activation of apoptotic effector proteins and the suppression of Bl-2 family activity [28]. The stimuli-induced increase in activity is multifaceted, with evidence of transcriptional, post-transcriptional and post-translational modifications all shown to play a role in triggering intrinsic apoptotic cascade [29]. The activation of BH3-proteins leads to the dimerisation of Bak and Bax on the MOM mediating the cytosolic release of Cytochrome C (Cyto-C), Smac/DIABLO and the serine protease HtrA2/Omi [26,27]. Cytosolic Cyto-C then forms a multiprotein complex known as the apoptosome, consisting of Apaf-1 and the zymogenic procaspase 9. The formation of the apoptosome induces the autoproteolytic processing of procaspase 9, with the active caspase 9 then cleaving effector caspases downstream [26,30] (Figure 3).

The extrinsic apoptotic pathway is triggered through the interaction of death receptors belonging to the tumour necrosis factor (TNF) receptor superfamily, including CD95 (APO-1/Fas), TNF receptor 1 (TNFR1), TNF-related apoptosis-inducing ligand receptor 1 (TRAIL-R1) and TRAIL-R2, with their respective ligands. The prototypical receptor–ligand pair is CD95:CD95L, an important regulator of T cell contraction but also T cell activation [31]. The binding of CD95L induces a conformational change in the intracellular region of CD95 and subsequent formation of the death-inducing signalling complex (DISC). DISC is composed of Fas-associated death domain (FADD), the procaspase regulator c-FLIP, and the inactive caspase 8 and -10 zymogens. The oligomerisation of procaspase 8 leads to autoproteolytic processing, with active caspase 8 initiating the apoptotic cascade [26,30,32].

Of note, it is possible for cells to proceed through two distinct pathways following procaspase 8 activations, deemed type I and II, respectively [33]. Type I responses occur when the levels of active caspase 8 are sufficient to process caspase 3, leading to apoptosis directly. Alternatively, type II apoptotic responses involve the cleavage of the Bcl-2 family member Bib and subsequently leads to the dimerisation of Bak and Bax on the MOM, mitochondrial depolarisation and the release of Cyto-C into the cytosol [33,34] (Figure 3).

The impact of apoptosis on the maintenance of T cell function can depend on the nature of the immune response and the differentiation/activation state of the T cell [35]. Intrinsic apoptosis regulates both naïve and activated T cells; however, the nonapoptotic Bcl-2 family members play varying roles throughout T cell life. T_N_ cells express high levels of Bcl-2, with a rapid decrease upon TCR engagement, concurrent with a rapid increase in Mcl-1 expression [35,36]. While Mcl-1 has been shown to be critical for T cell survival at all stages of development, Bcl-2 and Bcl-xL are thought to serve distinct roles during T cell development [37].

Triggering of external apoptosis through CD95:CD95L interactions is critical in ensuring overactive T cells are removed from the circulation during a healthy immune function by activation-induced cell death (AICD). Intriguingly, ablation of the CD95:CD95L pathway has been shown to have little impact upon T cell contraction during acute infections but is instead essential for controlling T cell expansion in chronic antigen stimulation [38]. Constitutive exposure to CD95L also impairs DISC formation and leads to the appearance of CD95-resistant T cells with reduced AICD capacity [39].

CD95-induced apoptosis is one of the main ways that both CD4^+^ and CD8^+^ cytolytic T_EFF_ kill transformed and virally infected cells [40]. Almost all human tumours express CD95 and CD95L on their cell surface [40]. Upregulation of CD95L along with downregulation of CD95 promote tumour progression. In the "tumour counterattack" theory, a high level of CD95L on the tumour cell surface activates AICD in TILs [40].

Tumour cells also benefit from nonapoptotic functions of CD95 signalling. Cancer cells upregulating CD95/CD95L express chemotactic factors such as IL-8 and MCP1 [41]. Chemotactic proteins increase the recruitment of proinflammatory cells and create an inflammatory environment supporting cancer growth [42]. Activation of CD95 in apoptosis-resistant tumour cells results in the induction of pathways or a set of genes with a variety of roles in tumour progression. For instance, activation of CD95 is an inducer for NF-κB and all three major mammalian target of rapamycin (MAPK) pathways: ERK1/2, p38 and JNK1/2 [43]. These pathways have implications for growth, invasion, metastasis, resistance to apoptosis and cell cycle progression [44]. Moreover, the vast majority of reports have shown that upregulation of CD95L by cancer cells is an adverse prognostic marker for many solid tumours [40], and the elimination of CD95 or CD95L in cancer cells induces "death induced by CD95 or CD95L" [45].

The implications of CD95:CD95L pathway has also been investigated in CAR T cell therapy. CAR T cells use CD95L to lysis CD95 positive target cells. Hong et al. showed that CD30-CAR T cells killed their CD30^+^ target cells as well as CD30^-^ surrounding cells via a cell–cell contact-dependent CD95:CD95L interaction [46]. In addition, it is well known that T_M_ cells express CD95 as their marker, and high CD95 expression have been shown in T memory stem cells (T_SCM_) [47]. In another study, Klebanoff et al. showed that stimulating CD95 signalling using soluble trimeric CD95L elevated memory CAR T cell differentiation [48].

Recent reports suggest that CD95:CD95L signalling may play a role in the limited long-term persistence of CAR T cells in treating solid tumours [49,50]. Blockade of the signalling pathway has been shown to both increase CAR T cell persistence and number without adverse effects [49,50,51,52,53]. Inhibiting the CD95:CD95L pathway enhanced the CAR T cell therapy in vitro and in vivo. For example, inhibition of CD95 or CD95L translation via siRNA increased the persistence of anti-CD171 CAR T cells [49]. Blockade of CD95:CD95L either with a dominant-negative form of FADD or mAb, increases the number of CAR T cells without causing autoimmunity [50,51,53]. However, due to the vital role of CD95:CD95L pathway in the development of memory CAR T cells, modulation of CAR T cells by ablating CD95 signalling must be investigated with an abundance of caution. We have recently shown that Her2-CAR T cells overexpressing Mcl-1 exhibit high resistance to AICD-mediated by CD95L [54]. Since Mcl-1 acts at a later stage of AICD, induction of Mcl-1 expression to overcome CAR T cell loss could be considered as a future approach. An additional advantage of modulating Mcl-1 expression is the ability of Mcl-1 to enhance mitochondrial and energetic dynamics and its potential role in the generation of T_M_ cells [37,55].

## 5. Mitochondrial Function during T Cell Activation and Differentiation

The initiation of T cell activation results in the reprogramming of cellular metabolic processes in order to meet the bioenergetic and biosynthetic demand. Sustained TCR signalling, and costimulation, are required to initiate metabolic changes to facilitate proliferation, differentiation and production of effector molecules such as cytokines [56].

T cell activation results in a shift from OXPHOS and FAO with the promotion of the anabolic pathways of glycolysis and glutaminolysis within T cells [57,58]. This is facilitated through the engagement of the TCR to a recognised antigen; this engagement must be both high affinity and sustained to initiate downstream activation pathways [59]. The costimulation mediated by CD28 is also needed to avoid senescence, and drive the metabolic shift toward anabolic pathways needed for activation [57]. TCR engagement phosphorylates receptor-proximal tyrosine kinases that in turn activate phospholipase Cγ (PLC-γ). PLC-γ mediates two pathways, one through Ca^+^ flux and the other through protein kinase C (PKC) activation. An increase of Ca^+^ through its release from the endoplasmic reticulum stimulates the phosphatase calcineurin, which dephosphorylates the transcription factor NFAT. This allows for NFAT nuclear translocation to promote the expression of genes associated with T cell activation such as the (mTORC)-1 and mTORC2 [57,58]. PKC activates the mitogen-activated protein kinase MAPKs family, which also increases the rate of ribosomal synthesis within activated T cells [60].

The activation of mTORC2 activates AKT, which increases expression of GLUT1, a glucose transporter. Increased GLUT-1 expression increases glucose uptake to fuel glycolysis, thereby facilitating cell growth and proliferation [12,17,57]. Glycolytic impairment due to lower GLUT-1 expression in CD8^+^ T cells obtained from chronic lymphocytic leukaemia (CLL) patients was suggested to contribute to dysfunctional CD8^+^ T cells [61,62]. Increases in intracellular glucose levels further induce the differentiation of T helper 1 (Th1) and T follicular helper (Tfh) cells [63]. However, an increase in glycolytic metabolism results in an elevated level of glycolytic byproducts such as lactate. To compensate for increases in intracellular lactate concentrations, activated T cells increase expression of the monocarboxylate lactate transporters (MCTs), which regulates lactate influx and efflux [64]. Increases in cellular lactate can lead to inhibition of glycolysis in effector cells [65,66], whilst regulatory T (T_reg_) cells are able to oxidize lactate to pyruvate, therefore, leading to an increase in T_reg_ cells [67]. Inhibition of lactate dehydrogenase (LDH) was shown to increase the importation of pyruvate into the TCA, promoting metabolic programming in CD8^+^ T cells with higher antitumour activity T_SCM_ phenotypes [68]. Tumour cells also possess a highly glycolytic metabolism, exporting high levels of lactate into the TME, resulting in impairment of effector cell differentiation and function, whilst creating a favourable environment for T_reg_ cells to further the immunosuppressive environment.

The increase of glycolysis also results in higher glycolytic intermediates such as pyruvate that is then converted to acetyl-CoA within the mitochondria via oxidation. Acetyl-CoA can then activate histone acetyltransferases (HATs), and act as an intermediate for the synthesis of fatty acids and amino acids [69].

mTOR activation results in glutamine anaplerosis via the upregulation of glutamine transporters such as ASCT2 [70]. Increases in cellular concentrations of glutamine result in fumarate accumulation, which has been shown to inhibit the action of histone demethylases that are involved in the differentiation of T cell subsets such as Th17 [71]. The ASCT2 transporter is highly overexpressed in many cancers such as melanoma [72], highlighting the competition for nutrients between malignant and immune cells within the TME. Therefore, glutamine metabolism may pose a serious therapeutic target not only for prevent tumour growth but also aiding in T_EFF_ cell proliferation and function.

ERK-MAPK activation leads to an increase in ribosomal synthesis [60]. T cell activation results in large increases in the expression of genes GAR1, NHP2, RRS1, EBP2, NIP7, HRAMT and dyskerin, which have all been shown to regulate rRNA processing [60]. Similarly, TCR-triggered ERK activation results in the phosphorylation of transcription factors responsible for rRNA synthesis. Ribosomal synthesis is crucial for the production of proteins needed for effective immune function. Inhibitors of mitochondrial ribosomal pathways have been shown to decrease mitochondrial protein synthesis and cytokine production leading to impaired immune function [60].

Mitochondrial mass and mitochondrial DNA (mtDNA) also have been shown to substantially increase by the first hour of activation [58]. mtDNA encodes numerous components of the mitochondrial ETC. Mitochondrial transcription factor A (Tfam) plays a distinct role in the regulation of these genes, their transcription and the stability of mtDNA. Tfam blockage results in an impaired mitochondrial respiratory chain and mtDNA depletion, and Tfam-knocked-out T cells are less proliferative compared to wild-type T cells [73]. Similar effects have been seen in T cells with dysfunction in complexes I, II and III caused by silencing the apoptosis-inducing factor (AIF) [74]. In addition, T cells that lack stomatin-like protein 2 (SLP-2) lean toward glycolysis with decreased proliferation and IL-2 secretion due to changes in the IMM that impair the functionality of complexes I, II and III [75,76,77].

During T cell activation, newly synthesised mitochondria are metabolically redesigned and are dependent on a one-carbon metabolic pathway, which is comprised of three interlinked reactions: the folate cycle, the methionine cycle and the trans-sulfuration pathway. These reactions provide one-carbon methyl units needed for biosynthesis of amino acids, phospholipids and nucleotides but also involved in maintaining redox homeostasis. The conversion of folate to tetrahydrofolate (THF) is the key step of one-carbon metabolism. Addition of a one-carbon methyl unit to THF results in the generation of 5,10-methylene-THF, which is a central metabolite needed for the continuation of many metabolic pathways including the methionine and trans-sulfuration pathways. Generation of 5,10-methylene-THF is an NADPH dependent process and is facilitated by the enzyme SHMT2. This conversion is the most critical step of the one-carbon pathway, inhibition of SHMT2 activity has been shown to diminish T cell proliferation and lifespan [78]. To compensate for the high consumption of NADPH the one-carbon pathway also generates NADPH though conversion of 5,10-methylene-THF to 10-formyl-THF, resulting in the conversion of NADP^+^ to NADPH.

NADPH is a key regulator of reactive oxygen species (ROS) and therefore the consumption and generation of NADPH/NADP^+^ during metabolic processes such as OXPHOS and glycolysis must be balanced to maintain effective cell function and prevent ROS induced apoptosis [79]. To protect the cell from ROS generated during metabolism, glutathione (GSH), one of the most abundant antioxidants within cells, is produced [79]. Production of GSH is dependent on glutamate uptake into the cell where glutamate cysteine ligase combines glutamate, glycine and cysteine derived from the trans-sulfuration pathway to form GSH. The detoxification of ROS species by GSH results in the generation of glutathione disulphide (GSSG), a reversible reaction dependent on NADPH, highlighting its role in maintaining redox homeostasis [79,80].

Increased glutaminolysis during T cell activation, as mentioned previously, not only provides increased ATP generation but also provides essential precursor metabolites for biosynthetic pathways including the synthesis of GSH, which facilitates redox homeostasis during T cell activation. Previous research by Gaojian et al. [81] illustrated that glutamine derived glutamate was a key precursor in the de novo synthesis of GSH and furthermore that glutamine catabolism directed T cell lineage fate, in particular that glutamine deprivation skewed differentiation toward T_reg_ phenotypes [82,83]. Decreased glutamine levels within the TME not only impacts T cell energy generation but also the ability for T cells to maintain redox homeostasis needed to ensure effective cytokine production and cell proliferation and lineage differentiation [83,84].

The rate of protein, nucleic acid and lipid biosynthesis is increased within activated T cells when compared to T_N_ cells in order to maintain high levels of cell division. Increases in biosynthetic activities can be attributed to increases in glycolytic intermediates, a result of increased glycolysis within activated T cells as mentioned above. Hexokinase (HK) isoforms are mediators for the rate limiting step of glycolysis, the conversion of glucose to glucose-6-phoshpate (G6P) [58,85]. Upon T cell activation there is a significant increase in the levels of HK1 and HK2 isoforms, shown to have the highest level of catalytic activity [86]. G6P can be dehydrogenated in the pentose phosphate pathway (PPP) to produce NADH for use as both a reducing agent in the synthesis of fatty acids and a cofactor in the synthesis of nucleotides for DNA replication. Dwindling glycolysis capacity by blocking HK2 favours T cell expansion [58], without affecting CD8^+^ T cell activation through TCR-CD28 stimulation [58,87]. HK2 knock-out causes a severe antitumour activity in CD8^+^ T cells by overexpressing PD-1 and Tim3 [87]. Interestingly, HK2 deletion seems to be dispensable for CD4^+^ T cell responses against viral infection [85,88] but not for CD8^+^ responses [87]. However, the long-term efficacy of T cells that no longer possess a key metabolic gene is difficult to predict. It is difficult to elucidate whether the effects observed after deletion of HK2 are direct or indirect due to lack of HK2. Besides, it has been shown that HK1 could serve as a compensatory gene in the absence of HK2 [88]. Hence, further studies using small molecules or knockdown HK1/HK2 are needed to clarify the HKs function in T cells.

Mitochondrial-derived acetyl-CoA concentrations also increase during activation due to increased pyruvate levels; another glycolytic intermediate increased upon activation [57]. During proliferation, glycolysis-derived pyruvate feeds into the TCA, where it is converted to acetyl-CoA and then citrate within the mitochondria. This citrate is then exported to the cytosol, where it is catalysed to cytosolic acetyl-CoA used in fatty acid synthesis and made available for the mevalonate pathway [89]. Citrate export is an example of how the mitochondria maintain balance during T cell expansion, as without activation induced increases in glutamine oxidation and glycolysis, citrate export would result in TCA stalling [89]. Instead, imported glutamine can be converted into α-ketoglutarate to enter the TCA to compensate for the lack of citrate conversion. Interestingly studies have shown excess α-ketoglutarate can undergo reductive carboxylation within the cytoplasm to form citrate and subsequently acetyl-CoA to also feed the mevalonate pathway [90]. The mevalonate pathway involves the condensation of acetyl-CoA to hydroxy-3-methylglutaryl-CoA (HMG-CoA). Through a cascade of anabolic conversions, farnesyl pyrophosphate (FPP) is formed and is a substrate for the synthesis of cholesterol, steroids, dolichol and ubiquinone [89].

T cell activation increases the expression of the genes, which encode HMG-synthase and reductase, both of which are needed for the initiation of the mevalonate pathway [89]. The role of cholesterol in T cell activation and its relation to solid cancers has not been fully investigated. Studies have shown that inhibition of ACAT1, a cholesterol esterification enzyme, has led to increased cellular cholesterol that has, in turn, increased CD8^+^ function through increased TCR clustering at the plasma membrane [91]. In contrast, other work has shown that increased cellular cholesterol has inhibited glycolysis, and increased expression of inhibitory markers resulting in exhaustion of TILs [92]. Thus, the exact role cholesterol metabolism has in T cell activation is not yet fully understood.

Overall mitochondrial metabolic pathways are reprogrammed during T cell activation away from OXPHOS and FAO and instead toward anabolic pathways such as glycolysis and glutaminolysis. The balance of intermediates produced within these pathways enables the biosynthesis of macromolecules needed for T cell activation. After antigen clearance, most T_EFF_ cells undergo contraction, while a small number of T cells differentiate to long-lived T_M_ cells. Activation of FAO metabolic program is necessary for T_M_ differentiation, long-term survival and enhanced response to the second antigen encounter [22]. It has been shown that the enhanced response in T_M_ cells compared to T_EFF_ cells is linked to the mitochondria mass, upregulation of genes involved in FAO and their higher spare respiratory capacity (SRC) [17,22]. The impact of the TME by disrupting and exploiting these processes promotes tumorigenesis by impeding immune metabolic pressures resulting in an inadequate immune response against the malignancy.

## 6. Mitochondrial State in TILs and Exhausted T Cells within TME

Solid tumours remain the greatest challenge to ACT, with limited responsiveness to a raft of currently available cellular therapies. In a large part, this is owing to the TME, which represents a highly immunosuppressive, physically obstructive and nutrient-depleted environment that severely limits the ability of ACT to generate effective antitumour responses. The TME also includes infiltrating immune cells, tumour stroma, blood vessels and soluble factors that all act to limit tumour immunity.

The presence of TILs within tumour masses is a known positive prognostic factor for the response to immunotherapy. However, certain TIL populations show a functionally exhausted phenotype and drastically reduced effector function, allowing for tumour escape. [11]. In addition to TIL, a range of T cell populations are present in the TME that suffer similar functional exhaustion that is thought to play a key role in the ability for malignant cells to outgrow and cause disease. As with the endogenous immune response, ACT approaches using CAR T cells for treating solid tumours face the same obstacles.

A range of studies has shown that this functional insufficiency and exhausted phenotype can be linked to mitochondrial dysfunction [93,94]. As previously described, a number of metabolic processes play important roles in maintaining REDOX balance within T cells [79,81,84]. Recent studies have indicated that tumour-localised T cells can show a loss of redox balance leading to the development of a functionally exhausted phenotype. Vardhana et al. showed that mitochondrial insufficiency in T cells exposed to chronic antigen leads to an increase in the NADH/NAD^+^ ratio [94]. The increased oxidative stress was linked to the expression of T cell exhaustion genes and an inability of these cells to undergo proliferation or self-renewal [94]. While this study showed that treatment of exhausted T cells with antioxidants could partially restore functional capacity, studies examining the efficacy of anti-PD-1 therapy have shown contrary results when examining the role of ROS in improving therapeutic outcomes [95,96,97]. Alternatively, a CAR T cell construct coexpressing catalase was shown to reduce intracellular oxidative stress and outperform traditional CAR T cells [98]. These results indicate that the role that ROS play in the functioning of T cells within the TME is poorly understood and will remain an area of active research.

Tumour cells show augmented metabolism to fuel their rapid proliferation, leading to markedly lower levels of a range of crucial nutrients in the TME than healthy tissue. The nutrient restrictions faced by T cells within the TME contribute to reduced effector function, proliferation and differentiation. Glucose is an essential nutrient required for the rapid generation of ATP to support the rapid proliferation of activated T_EFF_, and the production of cytokines for effective immune activity. Activation of tumour-specific T cells in glucose low concentration leads to hyporesponsiveness, with decreased expression of genes associated with cell cycle progression, effector function and cytokine production [8,11,99]. Glycolytically active T cells may be pushed towards an unsustainable metabolic program in which the ability to produce ATP through glycolytic metabolism is unable to satisfy cellular requirements. Glucose-deprived CD8^+^ T cells show an increased AMP:ATP ratio, leading to the activation of AMP-activated protein kinase (AMPK) and subsequent shift towards anabolic metabolism in an attempt to maintain viability and regulate energy homeostasis [12,17,21]. Additionally, the activation of AMPK in nutrient-poor conditions has been linked to the senescence of T cells. AMPK is known as the key element in the regulation of FAO [12,17,71,73,89]. In normal conditions, the differentiation toward T_M_ cells is partially linked to FAO, which is accomplished through AMPK and lipolysis.

In addition to glucose, glutamine is also scarce in the TME and serves as a key regulator of the function of activated T cells. Glutamine depletion reduces the ability of activated T cells to produce IL-2 or IFN-γ, and provoking a drastic reduction in activation mediated proliferation. Coupled with a loss in key effector functions, reduced glutamine impacts upon CD4^+^ T cell differentiation and further potentiates the TME [70]. Deficiencies in the ASCT2 glutamine transporter blunt T_N_ cell differentiation from Th1 to Th17 cells, but still maintain T_reg_ differentiation. However, downregulation of glutaminolysis regulates the conversion of Th1 toward T_reg_ cells [70,90]. Therefore, a lack of glutamine prevents T cells functioning correctly in the TME, and stimulates the emergence of regulatory T cell subtypes [100]. Recently Leone et al. [82] developed an inert glutamine antagonist (JHU083), which is only activated upon cleavage of the prodrug through enriched enzymes in the TME. They showed that conditional activation of JHU083 suppresses tumour cell growth while boosting the CD8^+^ T cell proliferation, activation and T_M_ differentiation. The combination of JHU083 with anti-PD-1 augmented the antitumour activity of TILs in vivo [82].

A hallmark of the TME is hypoxia generated by heterogeneous and ineffectual vascular networks and aberrant tumour cell metabolism. Hypoxia leads to a range of alterations in cellular function, including an increase in glycolytic metabolism and a decrease in proliferation [12,17,21]. The hypoxic response is mediated primarily through the action of the hypoxia inducible factor (HIF) family, with hypoxia leading to increased activity through stabilisation of HIFs. Hypoxia leads to the recruitment of T cells to the TME in addition to a vast array of potentially immunosuppressive cells to the TME including myeloid-derived suppressor cells (MDSCs) and macrophages [11,101]. The binding of HIF-1 to HIF regulatory elements induces the upregulation of PDL-1 in macrophages, MDSC and tumour cells [11,101]. Additionally, HIF signalling increases the expression of the coinhibitory receptor CTLA-4 on CD8^+^ T cells [101]. The upregulation of these coreceptors negatively impacts the ability of activated T cells to undergo glycolysis, and instead switches metabolic function towards FAO and reduces IFN-γ production [101]. Increased HIF-1a expression and encouragement of FAO are vital processes that allow the survival of T_reg_ in the TME compared to alternative T cell subtypes that show high glycolytic commitment [101]. Irrespective of the evidence outlined above, HIF signalling has been shown in some circumstances to potentially increase the cytotoxic capacity of CD8^+^ T cells [102]. However, these studies may not fully represent the encounter of CD8^+^ T cells with tumour antigen in the context of TME hypoxia [103].

The range of inhibitory immune cells present within the TME, including T_reg_ cells, produce a milieu of soluble factors that serve to negatively regulate the function of TILs. At the centre of these suppressive factors is TGF-β, secreted by MDSCs, macrophages, T_reg_ and tumour cells within the TME [11]. The impact of TGF-β upon the T cell function is distinct in T_N_ and T_EFF_ cells, with T_N_ cell differentiation augmented while TEFF show impaired proliferation and cytokine expression [104]. Blockade of TGF-ß in a murine tumour model has been shown to improve tumour regression and enhance the function of cytotoxic lymphocytes [105]. Accordingly, CAR T cells have been engineered to allow for resistance or subversion of TGF-β signalling. CAR T cells harbouring a knockout of the endogenous TGF-β receptor showed improved effector function and increased tumour elimination [106]. Additionally, redirecting CAR T cells to recognise soluble TGF-β converted the immunosuppressive effect of TGF-β, whilst also allowing for neighbouring immune cells to be protected from TGF-β induced T_reg_ differentiation [106,107].

## 7. Strategies to Improve CAR T Cell Therapy by Metabolic Reprogramming

Increased interest in the metabolism’s role in cancer progression and the impact on the effectiveness of immunotherapeutic treatments have led to the development of new therapies that aim to manipulate these metabolic processes increasing the efficacy of treatment. It is well-known that there is a tug of war between tumour cells and TILs for nutrition with a few exceptions, such as CLL, which lowers glucose uptake in TILs, not due to nutrition competition but instead, it was proposed to result from the lower expression of nutrition transporters on the cell surface of CD8^+^ TILs [61,62].

**Figure 4:**
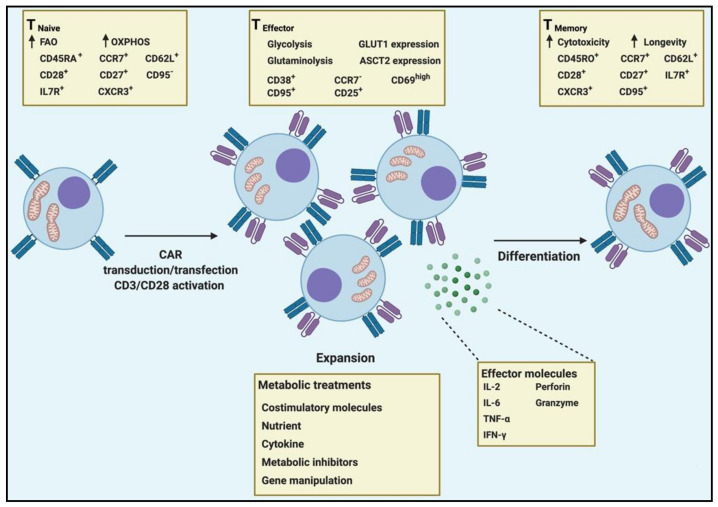
Metabolic interventions to reprogramme CAR T cell metabolism. T_N_ transduced/transfected with the CAR construct, following which they are stimulated with CD3/CD28 to generate T_EFF_ cells capable of tumour targeting and killing. In contrast to physiological T cells that undergo contraction after the expansion phase, CAR T cells are expanded for a period of around two weeks during which they can be metabolically reprogramed toward long-lived T_M_ cells or be acclimated to the nutrient profile of the TME.

In the last decade, strategies to overcome T cell hyporesponsiveness have mostly focused on the use of immune checkpoints (ICP) blockades such as anti-PD-1, anti-CTLA-4 and anti-LAG-3 [108]. Although using ICP blockade has shown promising results in clinical trials for the treatment of certain malignancies and adverse effects, termed immune-related adverse events (IRAEs), have also been reported [109,110]. In addition to cytokines (e.g., TGF-β and IL-10) and ICP, metabolic stresses on TILs confer a significant immunosuppressive effect on T cell function and differentiation. The disorganized vascular system within TME leads to low perfusion of nutrients and the accumulation of metabolic wastes such as adenosine, prostaglandin E2 (PGE2), lactate, vascular endothelial growth factor, phosphatidylserine, high extracellular K^+^ levels, hypoxia and free radicals [13]. Therefore, manipulating the metabolic pathways that armoured CAR T cells to encounter such a hostile environment emerge as worthwhile therapeutic strategies (Figure 4).

### 7.1. Costimulatory Molecules

The inclusion of costimulatory domains within CAR T cell constructs was one of the initial approaches used it improve CAR T cell efficacy. CD137 (4-1BB) is a potent costimulator of T cell proliferation and expansion and can improve T cell mitochondrial biogenesis and metabolism. Second and third-generation CAR T cells with 4-1BB costimulatory domains have shown superior metabolic capacity, enhanced antitumour activity, persistence and a higher number of memory phenotypes [2,3,111,112]. The increased metabolic function because of 4-1BB inclusion can be attributed to improvement in mitochondrial mass, biogenesis, mitochondrial membrane potential (ΔΨm), fusion, activation of the p38-MAPK pathway [111] and depends on OPA-1 [112]. CAR T cells using 4-1BB costimulatory molecules have been shown to persist for four years in patients with acute lymphoblastic leukaemia (ALL) [113].

CD28 is the most prevalent costimulatory domain used in CAR T cell therapy. It has been shown to be effective in the induction of glycolysis and differentiation toward effector memory phenotypes [114]. Dysfunctional TILs from CLL patients showed an impaired ability to induce glycolysis, the main metabolic program of TEFF cells [61]. A comparison of CD28 and 4-1BB costimulatory molecules showed that CAR T cells with 4-1BB have longer persistence as they tend to have a higher number of T_CM_ phenotypes [114]. However, such differences were not seen in patients with relapsed/refractory B cell leukaemia [115]. Moreover, it is worthwhile noting that the importance of CAR T cell persistence may also be cancer dependent [2,3]. Therefore, the type of cancer should be considered when choosing the costimulatory molecules in designing the CAR constructs.

Several strategies have been developed to convert the negative effects of immunosuppressive factors into the beneficial effects of costimulatory molecules. One approach involves the use of the chimeric costimulatory molecules termed “costimulatory converters” [116]. These are fusion proteins encompassing the extracellular domain that recognise immunosuppressive molecules, such as PD-1, fused to an intracellular domain of a costimulatory molecule such as CD28 or 4-1BB [117]. Another technology for the avoidance of CAR T cell immunosuppression uses bispecific T cell engagers (BiTEs). BiTEs are recombinant proteins consisting of two distinct scFvs coupled by a short flexible linker [118]. One of the scFv is specific to a TAA on the tumour surface, while the other recognises the CD3ɛ/γ heterodimer on T cells, enabling CAR T cells coexpressing the BiTE to engage with two antigens. This is of particular benefit when one of the TAA is heterogeneously expressed on the tumour surface [118].

### 7.2. Nutrition-Restricted Media

T cells are heterogeneous and plastic, facilitating the conversion to different subtypes and allowing adaptation to new environments. The plasticity enables them to respond to fluctuations of nutrients in the environment and adjust their metabolism accordingly. Shortly after T_N_ cells recognise an antigen, they differentiate to TEFF cells with specific metabolic characteristics and functional effects. Mimicking the TME metabolic stresses during the ex vivo expansion is a promising strategy to allow CAR T cells to adjust their metabolism to TME [14]. Evidence in favour of this approach includes studies that expand CAR T cells in restricted media. For instance, inhibition of glutamine metabolism via culturing T cells in glutamine-depleted medium or the addition of glutamine-metabolic inhibitors (e.g., AOA, L-Don EGCG) enhances the antitumour activity of CD8^+^ T cells and their trafficking in mouse models [119]. Expanding the CAR T cells in glucose-restricted media augments the CAR T cell function and T_CM_ phenotypes [120]. To make this strategy clinically practical, a metabolic atlas for all human tumours need to be developed. Finally, the nutrient restriction concept also appears to contrast with well-documented data concerning the suppression of T cell function by nutrient starvation [121].

### 7.3. Cytokines

Generally, after transduction or transfection, CAR T cells will be expanded in media supplemented with one or two γ chain (γc) cytokine families to facilitate CAR T cell expansion, survival and differentiation. So far, γc cytokines that have been used in manufacturing CAR T cells include IL-2, IL-7, IL-15, IL-21 and IL-9 [122]. Aside from the biological functions, γc cytokines also impact the metabolism of CAR T cells [122]. IL-2 seems to encourage anabolic metabolism necessary for T_EFF_ development through activation of PI3K and mTOR signalling [12]. IL-7 promotes FAO metabolism in CAR T cells through increased expression of glycerol transporters and triglyceride (TAG) synthesis [123]. IL-15 also fosters FAO metabolism through upregulation carnitine palmitoyl transferase and uptake of fatty acid precursors [14]. Similar effects have been observed for CAR T cells treated with IL-21 and IL-9 with enhanced FAO metabolism and higher mitochondrial fitness [124].

Expanding CAR T cells in a combination of IL-7, IL-15 or IL-21 leads to metabolic reprogramming from aerobic glycolysis towards FAO and promotes mitochondrial fusion and fitness. It also reduces the expression of exhaustion markers and increases the number of T_SCM/CM_ population. However, concentration, combination and duration of treatment should be carefully considered. CAR T cells grown solely in IL-15 had elevated mTORC1-p38 MAPK activity and higher T_SCM_ phenotypes compared to the combination of IL-7 and IL-15 [125]. Furthermore, continuous exposure to IL-15 can lead to NK cell exhaustion [126], although such an effect has not been reported in T cells. Moreover, continuous stimulation with IL-7 has been shown to induce “cytokine-induced cell death” (CICD) by elevating the level of active caspase-3, Fas, FasL and proapoptotic protein Bim [127].

### 7.4. Metabolic Inhibitors

The advantages of using metabolic inhibitors to reprogram metabolic pathways include the enzyme specificity and the ease at which concentration and time of exposure can be controlled. As mentioned before, the PI3K-AKT pathway increases glycolysis, leading to terminally differentiated and exhausted T_EFF_ cells [12]. Ex vivo culture of CAR T cells with 10 μM of LY294002 (PI3K inhibitor) yielded a higher number of T_SCM/CM_ cells and dramatically improved the efficacy of CD33 CAR T cells in vivo [16]. Interestingly, anti-BCMA CAR T cells cultured in IL-7 and IL-15 failed to control tumour growth in vivo, while CAR T cells preconditioned with IL-2 and PI3K inhibitor demonstrated long-term and complete remission in the mouse model [128]. Similarly, Akt inhibition in CAR T cells promotes FAO metabolism, dampens glycolysis pathways, reduced glucose uptake, lactate production and downregulating pro-apoptotic genes (BAX and BAD), whilst encouraging T_CM_ differentiation [9,15,129]. Lastly, using inhibitors against glycolytic mediators such as HK isoforms also improves persistence, effector function, T_M_ development and CAR T cell efficacy in vivo [9,130,131]. Taken together, ex vivo supplementing CAR T cells with metabolic inhibitors is a feasible approach in manufacturing fitter CAR T cells for solid tumours.

### 7.5. Gene Manipulation

Advances in molecular biology have fuelled CAR T cell therapy, allowing more sophisticated genetic constructs to function or differentiate toward desire cell products. Up-/downregulation and knock-in/-out of target genes in CAR T cells have been widely used to improve CAR T cell performance. The expression of genes involved in a particular metabolic pathway can be modulated using constitutive or inducible systems. The constitutive expression can be achieved using dual-promoter systems [132], bidirectional promoters [133], 2A self-cleaving peptides [52] or adding internal ribosome entry site (IRES) sequences [134]. For controlled expression over gene-of-interest (GOI), tetracycline (Tet)-On/Off system [135] or endogenous inducible promoters (e.g., IL-2 minimal promoter) [136] can be employed. The use of base-editing using clustered regularly interspaced short palindromic repeats (CRISPR)-Cas-based technologies can introduce mutation(s) in the coding region of DNA, resulting in mutant enzymes that no longer bind to their natural ligand allowing for more precise control of genes with minimal side effects [137].

Amino acid uptake by tumour cells limits the availability of amino acids for TILs, which are vital for T cell function and survival. The manipulation of amino acid trans-porter expression can be used to increase uptake of critical nutrients whilst also allowing for removal of metabolites, which might otherwise inhibit effector function. T cells express a low level of arginosuccinate synthase (ASS) and ornithine transcarbamylase (OTC), making them vulnerable to arginine deprivation in TME. Exogenously overexpression of ASS and OTC enzymes in CAR T cells, using 2A self-cleaving peptides, significantly enhanced CAR T cell performance in vivo without negative impact on exhaustion and cytotoxicity of CAR T cells [99].

Adenosine is the final byproduct of ATP consumption. It has been shown to accumulate in TME and suppress TILs function [11]. Adenosine binds to adenosine 2a receptor (A2aR) on activated T cells, where it promotes production and accumulation of cytosolic cyclic AMP (cAMP), which dampens T cell activation and cytotoxicity [138]. Downregulation of A2aR transcripts using shRNA, enhanced cytotoxicity, cytokine production and in vivo activity of anti-mesothelin-CAR T cells [139]. In another approach, Newick et al. used a decoy peptide to block the activation of protein kinase A (PKA) signalling [140]. In this study expression of a small peptide called RAID (regulatory subunit I anchoring disruptor) was incorporated into CAR T cells and showed that CAR-RIAD T cells were resistant to the immunosuppressive effects of PGE2 and adenosine [140].

Overall, whilst there is great promise in incorporating gene manipulation in CAR T cell therapy there are also disadvantages that limit the feasibility of the approach. Firstly, although several tools have been used to control GOI expression, the basal expression or immunogenicity of transactivators in such systems restrain their performance [135,141]. Secondly, the expression patterns of genes within T cells are still largely unknown, and uncertainty surrounding the extent of the interactions between transgenes and their endogenous regulators. Continuous expression or inhibition of a gene may affect other pathways with unpredicted outcomes. Lastly, providing additional promoter or regulatory elements (e.g., IRES sequences) could negatively impact the viral packaging and transduction process, thereby reducing the efficiency of CAR T cell manufacturing and adding additional cost to an already expensive therapy. Therefore, whilst gene manipulation is a valuable approach for the study of metabolic pathways through the regulation of GOI within a CAR T cell system in vitro and in vivo, further understanding of the extent and control of metabolic processes is needed before it is a practical approach in the clinic.

## 8. Conclusions

Several factors in the TME restrain the potential application of CAR T cell therapy for solid tumours. To mount an effective and durable treatment, there have been substantial efforts to discover the metabolic properties of highly effective CAR T cells. CAR T cells from patients with sustained remission have higher T_N/SCM_ and T_CM_ phenotypes with FAO/OXPHOS metabolism and higher mitochondrial mass. Numerous studies support the idea that reprogramming metabolic activity and mitochondria during expansion is a feasible approach to obtain better clinical outcomes. The time frame from the isolation of a patient’s T cells, transduction with CAR construct, and ex vivo expansion prior to infusion into the patient provides a feasible metabolic intervention window. A variety of approaches can be used to reach this objective by utilising costimulatory domains, treatment with cytokines, pharmacological drugs and gene manipulation.

## Figures and Tables

**Table 1:** List of recent clinical trials for CAR T cell therapy.

Clinical Trial Identifier	Phase of Study	Start Date	Target Cancer	Target Antigen	CAR Structure and Specification
NCT04697940	I/II	2020	Relapse and refractory B-cell NHL	CD19 and CD20	Tandem dual Specificity targeting CD19 and CD20 CARs
NCT04503980	I	2020	Colorectal cancer, Ovarian cancer	MSLN	MSLN-CAR-T cells secreting PD-1 nanobodies
NCT04185038	I	2019	Wide range of brain tumours	B7H3	Autologous T cells lentivirally transduced to express a 2nd generation B7H3 CAR and EGFRt
NCT03618381	I	2019	Wide range of brain tumours	EGFR806	Autologous T cells that are lentivirally transduced to express 2nd generation EGFR806 CAR and EGFRt
NCT03500991	I	2018	Wide range of brain tumours	HER2	Autologous T cells lentivirally transduced to express a 2nd generation HER2 CAR and EGFRt
NCT03198052	I	2017	Lung Cancer	HER2, MSLN, PSCA, MUC1, Lewis-Y, GPC3, AXL, EGFR, Claudin18.2 and B7-H3	3rd generation CAR-T cells targeting HER2, Mesothelin, PSCA, MUC1, Lewis-Y, GPC3, AXL, EGFR, Claudin18.2, or B7-H3
NCT03618381	I	2019	Paediatric Solid Tumours	EGFR806, CD19 and HER22tG	Autologous T cells lentivirally transduced to express a 2nd generation EGFR806-EGFRt and a 2nd generation CD19-Her2tG
NCT03525782	II	2018	Non-small cell lung cancer	MUC1	Autologous anti-MUC1 CAR-T cells with PD-1 knockout
NCT04489862	Early I	2020	Non-small-cell lung cancer mesothelioma	MSLN	Autologous MSLN- CAR T cells secreting PD-1 nanobodies
NCT04581473	Ib/II	2020	Gastric adenocarcinoma/Pancreatic cancer/Gastroesophageal Junction Adenocarcinoma	Claudin18.2	2nd generation
NCT03356782	I/II	2017	Sarcoma/Osteoid Sarcoma/Ewing Sarcoma	CD133, GD2, MUC-1 and CD117	4th generation CAR T cell
NCT03916679	I/II	2020	Relapsed and refractory epithelial ovarian cancer	MSLN	CRISPR/Cas9 mediated PD-1 knocked-out
NCT03323944	I	2020	Pancreatic Cancer	MSLN	2nd generation/fully humanized/lentiviral transduced huCAR T-meso cells
NCT03198546	I	2019	Hepatocellular carcinoma	GPC3 and TGF-β	CD4^+^ T cells are genetically engineered to express TGFβ-CAR and secret IL-7/CCL19 and/or scFvs against PD1/CTLA4/Tigit; CD8^+^ T cells are constructed to express GPC3-DAP10-CAR with knockdown of PD1/HPK1
NCT02915445	I	2016	Nasopharyngeal carcinoma/Breast cancer	EpCAM	3rd generation
NCT01869166	I	2018	Unresectable/metastatic Cholangiocarcinoma	EGFR and CD133	2nd generation
NCT03198546	I	2017	Advanced hepatocellular carcinoma	GPC3 and TGF-β	3rd and 4th generation CART cells with/or without IL-7/CCL19 and/or scFv against PD1/CTLA4/Tigit in T cells knockdown of PD1/HPK1
NCT03818165	I	2019	Metastatic Pancreatic Carcinoma	CEA	Using pressure enhanced delivery device (PEDD) to increase the CAR T cell migration
NCT04650451	II	2018	HER2-positive Gastric cancer HER2-positive Breast cancer	HER2	Dual-switch using inducible coactivation domain MyD88/CD40 and an CaspaCIDe® safety switch

CAR, chimeric antigen receptor; NHL, non-Hodgkin lymphoma; MSLN, mesothelin; EGFRt, truncated epidermal growth factor receptor; HER2, human epidermal growth factor receptor 2; PSA, prostate-specific antigen; MUC1, mucin1; GPC3, glypican-3; scFv, single-chain variable fragment; PD1, programmed cell death protein 1; CTLA4, cytotoxic T-lymphocyte-associated protein 4; Tigit, T cell immunoglobulin and immunoreceptor tyrosine-based inhibitory motif (ITIM) domain; EPCAM, epithelial cell adhesion molecule; HPK1, hematopoietic progenitor kinase 1; CEA, carcinoembryonic antigen.
